# Skincare in Social Media: Analyzing Prominent Themes in Online Dermatologic Discussions

**DOI:** 10.7759/cureus.14890

**Published:** 2021-05-07

**Authors:** Aakash Reddy

**Affiliations:** 1 College of Natural Sciences, University of Texas at Austin, Austin, USA

**Keywords:** social media, reddit, skin health, online dermatology, skincare

## Abstract

Introduction: As the size and influence of online dermatologic communities have developed significantly in recent years, literature concerning the relationship between social media and dermatology grows more important. One community of interest is r/SkincareAddiction, a forum of over 1.2 million members on Reddit that serves as both a support group and discussion aggregator sourcing information from multiple social media platforms.

Methods: This study reports on the qualitative analysis of 300 highly engaged posts to identify prominent themes in online dermatologic discussions and determine the extent of multiplatform interaction between Reddit and other dermatology-relevant social networks.

Results: Results of an inductive analysis indicate discussions place an emphasis on communicating advice and improving general wellbeing, comprising 71% of measured posts. However, a large portion of this counsel lacks evidence and pseudoscientific recommendations are often accepted as factual. Additionally, 31% of entries were drawn from alternative social media sites.

Conclusion: Assessing the prevalent points of discussion in dermatology-relevant communities can inform clinical practice and reveal alternative methods to advance the delivery of care. As patients increasingly seek health advice and support online, developing digital literacy in common dermatologic trends offers a unique opportunity to improve communication, disseminate evidence-based counsel, and combat misinformation in both clinical and online settings.

## Introduction

A growing body of literature is emphasizing the constantly evolving relationship between social media and dermatology [1-5]. As platforms such as Facebook and Instagram are used more frequently by patients seeking health advice and support on dermatologic conditions, literacy in online dermatologic trends becomes more important to improve communication, combat misinformation, and improve patient health outcomes [3,4]. In particular, communication between dermatologist and patient has been established as being crucial for effective diagnosis and treatment [6].

Online dermatologic forums on Reddit, the self-styled “front page of the internet”, often discuss general skin care trends. One such message board is the subreddit titled r/SkincareAddiction where participants interact with large numbers of posts through commenting or voting. Boasting a community of 1.2 million anonymous members, the forum focuses on general skin health, cosmetic advice, and other dermatologic phenomenon [3]. Many of the most popular posts on r/SkincareAddiction are cross-posted from other social media platforms such as Twitter, Instagram, and Tumblr. Consequently, the site functions as a discussion aggregator sourcing content across the internet, indicating Reddit incorporates a diverse cast of participants from the online social sphere. This study intends to classify and analyze popular points of discussion and dermatologic trends on Reddit.

## Materials and methods

The framework approach uses a standardized procedure in thematic analyses of qualitative data and enhances methodological transparency by employing explicitly defined stages of analysis [7]. The approach in practice involved investigating the 300 most upvoted posts in the subreddit r/SkincareAddiction for patterns in text that were then inductively coded. Each code was categorized through an assignment to broader qualitative themes that were constantly refined through each stage of the process. Although some posts may be able to be classified in more than one code, posts were categorized into the one they most accurately represent as a whole. Usernames were not included to ensure the anonymity of posters was preserved.

The data was analyzed through three stages [7]. The first, data management, involved the identification and assignment of initial codes from posts in the subreddit to a coding matrix. The second, descriptive accounts, involved the refinement of initial codes and themes and the formation of conceptual connections. The final stage, explanatory accounts, involved drawing general conclusions, relating associations between themes, and reflecting on the original data. In the first stage, the data was catalogued into a coding matrix comprised of five columns: post text, post description, In-vivo code, preliminary impressions, and initial code. Post descriptions and In-vivo codes (phrases taken from post text) were used to illustrate key concepts to ensure the voice of the original poster was being represented accurately [7]. Relevant initial codes were then grouped together into general themes in a coding index. Throughout the investigative process, both coding models were constantly refined to reflect the emergence of new patterns and associations. Increasingly nuanced depictions of the data developed as new insights were drawn between initial codes. In the final stages, broader concepts were developed into conclusions that reflected the data as a whole. Final themes were evaluated against initial data to ensure an accurate representation of the original text and prevent misinterpretation during the refinement process.

Additionally, sample size is considered sufficient when the point of saturation has been reached. Inductive thematic saturation for qualitative data is dependent on the number and frequency of new codes. Redundant codes indicate saturation has been established [8]. Consequently, 300 posts offer a sufficient dataset to draw preliminary codes from the most engaged points of discussion in the subreddit.

## Results

Of the top 300 posts in the forum r/SkincareAddiction, four major themes emerged: i) advice on routines and products (33%), ii) general discussions of skin health and wellbeing (39%), iii) impact of skin ailments and their treatment options (14%), and iv) social concerns in relation to self-image, media representations, and the skincare community (14%). Results are listed in Table 1. Each theme emerged by classifying a total of twelve refined codes that were initially in-vivo descriptions of the original text. For posts that disseminated advice, the expressed intent was to improve mental health and general wellbeing, together comprising 71% of measured posts.

**Table 1:** Inductive coding of the top 300 posts in r/SkincareAddiction

Theme	#	Code	Code Description and Examples	Total
Advice	1	General	General product safety, hygiene recommendations, techniques to break harmful skin habits.	37
	2	Skincare Routine	Routine types and procedure, frequency of routine, description of personal regimen	23
	3	Product Recommendations	Suggestions in favor of and against specific skincare products and companies.	38
Skin Health	4	General Wellbeing	Emphasize mental health, community support and understanding, sharing narratives of improvement	52
	5	Showcasing Satisfaction in Personal Skin Health	Selfies displaying skin, before and after pictures depicting transformations in skin.	24
	6	Sharing Personal Struggles	Discussing frequent issues agitating community members, venting frustrations.	40
Effects on Skin	7	Acne	Treatment options, showcasing successful treatments, inquiries of product types.	25
	8	Scarring	General discussions of scarring, Acne scars, miscellaneous facial scarring.	6
	9	Sun	Sunburns, skin reactions to variable weather, expressing the necessity of sunscreen.	12
Social	10	Self-image	Factors influencing self-esteem, reflections on media representations of skincare	25
	11	Gender Bias	Discussions of gendered marketing strategies, relationship between gender identity and skin care.	11
	12	Community	Material interactions between members of the community, care packages for healthcare workers.	7
Total				300

Additionally, 31% of posts were cross-posted across multiple social media platforms. Of these, 44 are from Twitter, 43 are from Instagram, four are from TikTok, and three are from Tumblr. Posts from Twitter and Instagram were 47% and 46% of cross-posts respectively. On the other hand, posts from TikTok (4%) and Tumblr (3%) were a notably smaller proportion. The extent of the subreddit’s multiplatform interactions is recorded in Table 2. Ten posts directly referenced dermatologic practices such as by recommending a consultation, passing on advice from a provider, or disseminating social media posts by dermatologists.

**Table 2:** Posts from alternative platforms

Platform	Cross-posts	% of total posts	% of cross-posts
Twitter	44	15%	47%
Instagram	43	14%	46%
TikTok	4	1%	4%
Tumblr	3	1%	3%
Total	94	31%	

## Discussion

Online forums such as r/SkincareAddiction form tight-knit communities to express grievances, share personal stories, and develop unique points of discussion. As users credit the subreddit with significantly improving their knowledge of skin health, many posts take the form of counsel to others as a way to pass on shared knowledge. As members of online forums frequently consult social media for dermatologic information, advice posts may become more influential in the decision-making patterns of dermatologic patients [3]. While general advice was within an atmosphere of mutual learning, the advice in question varied widely from hygienic techniques (wash pillowcases frequently) to breaking habits (stop touching your face). However, advice relevant to skincare routines directly associated a disciplined routine with stability and organization. Regardless of the nature of the practice, simply having a skincare regimen done on a consistent basis was a regulatory force for many posters. For advice on skincare products and companies, some incorporated ethical concerns such as predatory corporations and labor abuse.

The largest number of posts were coded under general wellbeing and related skin health to security, happiness, and fulfillment. Members of the community argue their focus is beyond just appearance, indicating that skincare is a catalyst for general health and wellness. In particular, posters associate skin health with their mood as one poster expressed distress “when I wake up with a pimple”. Conversely, posts of individuals showcasing satisfaction with their skin were accompanied by positive remarks on their disposition (“self-esteem is back”). When posters feel frustrated, however, they are more likely to share their struggles, venting to the community. The subreddit serves as a support group through communicating problems and encouraging other members (“Your skin is NOT disgusting”).

When concerned with the specifics of skin health, posters emphasize the effects of particular ailments on their skin. Posts coded under 'sun' are generally discussions noting the utility of sunscreen and the dangers of uninterrupted sunlight. For example, one poster described their experience consistently applying sunscreen for years, noting how they felt significant improvements in physical appearance compared to when they had not used sunscreen at all. Posts coded under acne, however, tend to be of transformations (i.e. before and after pictures) and successful treatments. For some posters, acne was associated with depression ("defeated fungal acne… and depression”), reflecting the extensive literature associating acne with depression in dermatology patients [9-13]. Posts on scarring were also frequently related to acne, such as a request for advice on removing acne scars or a successful acne scar treatment. Many members of the community lamented the visual effects of both acne and acne scarring on their physical appearance. Posters discussing scarring also tended to depict transformations using before and after pictures, recognizing improvements in self-esteem [14,15]. Self-confidence can also be negatively affected through what posts identify as unrealistic beauty standards in media (“it’s just a whole lot of…photoshop”) and use the forum as a mechanism to combat the pervasiveness of media representations. The community condemns marketing campaigns that display heavily edited photos of skin. Gendered marketing strategies often play into these depictions as numerous posts reflect on how their gender informs interactions with skincare companies and products [16]. In response, the subreddit rallied around building a skin positive community with the goal of showcasing imperfections and encouraging the reconstruction of a healthy self-image. Reflecting a prior study on online dermatologic communities, shared struggles appear to link the subreddit’s members together and strengthen communal bonds through mutual aid and support [17]. Of cross-posts from alternative social media platforms, the majority were from Twitter and Instagram, sites that are frequently used by dermatology-relevant groups [18]. The large reach of Reddit incentivizes the accumulation of discussions from a wide range of sources. Consequently, just under 1/3 of the top 300 posts are from supplementary social networks.

Due to the nature of qualitative analysis, methodological limitations include subjectivity and lack of transparency [19]. In addition, posts from mass communication platforms are not primed for research. As a result, it is important to approach the coding matrix with caution to retain a strong basis in the text and avoid overstating conclusions. The framework approach was used to counter these limitations by highlighting the procedure of qualitative coding, identifying the stages of analysis, and using in-vivo text as initial codes.

## Conclusions

The vast size and influence of r/SkincareAddiction offer a compelling rationale for improving digital literacy in online dermatologic trends. The forum’s accumulation of posts from multiple social media platforms animates the subreddit as a vehicle for guidance, support, and dialogue. Although these resources can educate patients, misinformation regarding treatments and product sourcing can be equally rampant. Through accounting for popular points of discussion, general inaccuracies, and common themes on dermatologic social media, the information explored in this project can aid in developing a shared physician-patient vocabulary in online and clinical settings. In consideration of previous literature, future research can emphasize distinctions between dermatologic social media platforms and assess the comparative relevance between online discussions and successful clinical outcomes.
